# Maternal satisfaction on breastfeeding among paediatric outpatient mothers

**DOI:** 10.6026/973206300210910

**Published:** 2025-05-31

**Authors:** Vijayasamundeeswari P., Nalini S., Sarasapharina G.J., Sujatha N.S.

**Affiliations:** 1 Department of Paediatric Nursing, Sri Ramachandra Institute of Higher Education and Research, Porur, Chennai, India; 2 Department of Psychiatric Nursing, Sri Ramachandra Institute of Higher Education and Research, Porur, Chennai, India

**Keywords:** Maternal satisfaction, breastfeeding, mothers of infant, paediatric outpatient department

## Abstract

Breast milk is the optimal food for infants and hence early initiation of breastfeeding is crucial for successful breastfeeding. Therefore, it is of interest to assess the maternal satisfaction on breastfeeding among 150 mothers at Sri Ramachandra Hospital, Chennai, using a descriptive research design and convenience sampling. Participants included mothers who exclusively breastfed their infants and were willing to participate and assessed using the Maternal Breastfeeding Evaluation Scale. The majority of mothers were aged 26-30 years (56%), housewives (70%) and from nuclear families (50.7%); 66.5% showed a high level of satisfaction with breastfeeding. It should be noted that nurse educators play a vital role in promoting breastfeeding by educating mothers on its benefits for both the infant's development and the mother's well-being.

## Background:

The optimal nourishment for babies is breast milk, which offers numerous advantages. It contains antibodies that resist bacteria and viruses, as well as a relatively high level of secretory immunoglobulin (IgA) that prevents microorganisms from sticking to the intestinal mucosa Furthermore, it contains substances that hinder the growth of common viruses such as rotavirus and adenovirus [1, 2]. Successful exclusive breastfeeding depends greatly on correct early breastfeeding. There are ongoing campaigns to promote awareness about breastfeeding and emphasize its significance for both babies and mothers [3, 4]. Premature infants, *i.e.,* infants born before 37 weeks and their mothers represent a highly vulnerable group regarding breastfeeding [5, 6]. Premature babies are not developmentally mature and cannot be exclusively breastfed from birth like full-term babies [7, 8]. As a result, mothers and infants undergo a distinct and sometimes prolonged transition period from tube feeding to breastfeeding [9, 10]. The experience of breastfeeding varies among mothers of preterm infants, being perceived as positive and smooth for some and challenging for others [11, 12]. Numerous efforts have been made to improve this situation, taking into account the various factors that influence successful breastfeeding, including social, economic, cultural, political and personal factors [13, 14]. One aspect that could impact the duration of breastfeeding, yet has been inadequately explored in the literature, is women's satisfaction with breastfeeding [15, 16]. Therefore, it is of interest to to assess the maternal satisfaction on breastfeeding among mothers of infants attending the pediatric outpatient department at a selected hospital.

## Objectives:

[1] To assess the level of maternal satisfaction on breastfeeding among mothers of infant.

[2] To associate the level of maternal satisfaction with selected demographic variables.

## Methods:

A descriptive research design was adopted and the study was conducted in Sri Ramachandra Hospital Chennai. The sample size for this study was 150 and the samples were recruited by convenience sampling method. The inclusion criteria for the study were mothers who give exclusive breastfeeding to their infant and willing to participate in the study. Ethical clearance was obtained from institutional ethics committee. The instrument used for this study was Maternal Breastfeeding Evaluation scale. The collected data were analyzed using descriptive statistics.

## Sampling criteria:

## Inclusion criteria:

Mothers who,

[1] Gives exclusive breastfeeding to their infant

[2] Are willing to participate

[3] Can able to speak Tamil or English

## Exclusion criteria:

Mothers who

[1] Provides supplementary feed

[2] Are not feeding more than 1 month

[3] Are having child with congenital problems and any chronic illness

## Description of the instrument:

The instrument used for this study was Maternal Breastfeeding Evaluation scale. The data collection has two sections.

## Section-A:

It consists of selected demographic variables. Such as Age, Education, Occupation, Religion, Type of family, No of children, Place of Residence, Family income, Dietary pattern.

## Obstetrical history:

Mode of delivery, Source of information about breast feeding, Sex of the baby, Initiation of breastfeeding, Gestation age, Complication during antenatal period, Previous hospitalization of child , Abnormality in the breast.

## Section B:

Questionnaires consist of 30 questions about maternal satisfaction. Each items was assessed by using a 5 point Likert scale, ranging from 1 (strongly disagree) to 5 (agree).

It has 3 components;

[1] Maternal enjoyment / role attainment scale,

[2] The infant satisfaction / Growth subscale,

[3] Life style / maternal body image.

## Scoring and interpretation:

The following question was negative phrased items: 3, 5, 8, 13, 14, 15, 19, 22, 27, 28, 29. To transform (reflect) the scores, subtract each participant's rating from (*i.e.*, 1 becomes 5, 2 becomes 4, *etc.*) (Figure 1 see PDF).

[1] 74- 90 - Low level of satisfaction

[2] 91-106 - High level of satisfaction

## Results and Discussions:

## Demographic variables:

The sample comprised of 150 mothers. Out of 150 mothers, the demographic variables revealed that regarding age 56.0% belongs to 26-30 years, 7% belongs to 36-40 years. With respect to education; 46.7% as got high school education 30.3 % has got a graduation. The study revealed on occupation, 22.7% mothers were private sector working mothers. Regarding religion 65.3% were Hindu religion, 4.0% are in Muslim community. With regards to type of family 50.7% of mothers lived as a nuclear family, 49.3% of peoples lived in joint family. The data on number of children 44.7% of mothers had only one child. 51.3% of peoples lived in urban area. With respect to family income 50.7% had got income of Rs.5000-10000. Regarding to the dietary pattern, 66.7% of mothers are non- vegetarian. 5.3% mothers were mixed diet. With respect to mode of delivery 56.7% of mothers had a normal vaginal delivery, 42.7% of mothers had forceps delivery. Regarding source of information about breastfeeding 56.0% of mothers got information from family members, With respect to sex of baby 53.3% of mothers has got a boy child, 46.7%. Result with initiation of breast feeding 52.7% of mothers had started within 1 hour, regards to gestational age 50. % of mothers had delivered in < 37 weeks. With respect to complication during antenatal period 26.0% of mothers said yes. The study on previous hospitalization of child 28.7% of mothers said yes. Table 1 depicts that mean and standard deviation of the level of maternal satisfaction on breast feeding among mothers of infant mean value of 94.07 and SD of 6.604.

The maternal satisfaction on breastfeeding of mothers of infant were found to had statistically significant association between the religion, initiation of breastfeeding, complication during antenatal period at p<.001. This was supported by the study conducted to assess the breast feeding methods and satisfaction on feeding mothers in London. A total sample size was 560 were attained Maternal breast feeding assessment scale was used for the study. The findings revealed that positive correlation on initiation of breast feeding with an hour [17, 18]. The study revealed that with regards to maternal satisfaction on breastfeeding, Maternal enjoyment/Role attainment scale had the highest mean of 44.16 with standard deviation of 4.334 and Lifestyle maternal body image had the lowest mean of 24.75 with standard deviation of 2.627. The mean of overall 94.07 with standard deviation of 6.604.The data revealed that the mean of maternal satisfaction on breast feeding high satisfaction. The level of maternal breastfeeding methods were described at Mumbai. The sample size was 255 samples of mother Breast feeding techniques scale was adopted for the study. The study revealed that 56.7 %of mothers had high level of satisfaction 30.6 %participants with low level of satisfaction [19, 20]. The mean score for the Infant Satisfaction/Growth subscale was 25.15, indicating a moderate level of perceived infant satisfaction and growth among the mothers surveyed. This score suggests that while many mothers perceive their infants as content and developing appropriately, there may be variations influenced by individual and contextual factors. Previous research has highlighted the significance of maternal-infant bonding, breastfeeding experiences and overall maternal well-being in shaping perceptions of infant satisfaction and growth [21, 22]. A mean score of 25.15 reflects that, while most mothers perceive their infants as thriving, there may be underlying concerns regarding feeding adequacy, weight gain, or infant temperament. Studies have shown that factors such as breastfeeding difficulties, postpartum anxiety and limited social support can negatively influence maternal perceptions of infant satisfaction and growth [23]. Maternal perception of infant growth and satisfaction is a critical factor in early care giving behaviour and can influence long-term child health outcomes. Studies suggest that mothers with higher perceived confidence in their infant's growth are more likely to engage in responsive parenting practices, which contribute to optimal developmental trajectories [24]. The present study found that the mean score for the Lifestyle/Maternal Body Image subscale was 24.75, indicating a moderate perception of lifestyle satisfaction and body image among mothers. This finding suggests that while many mothers adapt to postpartum lifestyle changes and body image shifts, some may experience challenges in adjusting to these transformations. Maternal body image perception is known to be influenced by multiple factors, including postpartum weight changes, self-esteem, social influences and psychological well-being [25]. Studies have shown that postpartum body dissatisfaction is prevalent, particularly due to weight retention, physical changes and societal beauty standards [26]. Negative body image has been linked to higher levels of postpartum depression, lower self-esteem and decreased maternal confidence, which can indirectly affect maternal-infant bonding and overall well-being [27].

## Conclusion:

Mothers of infants generally have a high level of satisfaction with breastfeeding, with significant associations found between maternal satisfaction and factors such as religion, initiation of breastfeeding and complications during the antenatal period. Nurse educators should play a key role in educating mothers about the importance and long-term benefits of breastfeeding for both infants and mothers. Lactation nurses should provide guidance on effective feeding techniques, proper ways to hold the baby and maintaining hygiene during breastfeeding.

## Figures and Tables

**Table 1:** Mean and standard deviation of level of maternal satisfaction on breastfeeding among mothers of infant (N=150)

**S.no**	**Variable**	**Mean**	**Standard Deviation**
1	Maternal enjoyment/Role attainment scale	44.16	4.334
2	The infant satisfaction/Growth subscale	25.16	2.629
3	Life style/maternal body image	24.75	2.627
Overall mean and standard deviation		94.07	6.604
